# Student Perceptions of Secondary Education Teaching Effectiveness: General Profile, the Role of Personal Factors, and Educational Level

**DOI:** 10.3389/fpsyg.2019.00533

**Published:** 2019-03-11

**Authors:** Carmen-María Fernández-García, Ridwan Maulana, Mercedes Inda-Caro, Michelle Helms-Lorenz, Omar García-Pérez

**Affiliations:** ^1^Department of Education, University of Oviedo, Oviedo, Spain; ^2^Department of Teacher Education, University of Groningen, Groningen, Netherlands

**Keywords:** teaching, teacher effectiveness evaluation, adolescence, effect size, secondary education

## Abstract

The purpose of this study was to examine student perceptions of teaching behavior. Additionally the aim was to examine if teacher characteristics (educational level, gender, and teaching experience) could explain differences in student perceptions of their teachers. Teaching behavior was studied from the research on teaching and teacher effectiveness perspective. Secondary students (*N* = 7,114), taught by 410 teachers in Spain, participated in the study. Survey data were analyzed using non-parametric tests, Kruskal–Wallis, *U* Mann–Whitney with Bonferroni correction, and the analysis of effect sizes. Furthermore, a regression model was applied. Results showed that teaching behavior was perceived as sufficient to good, depending on the teaching behavior domain. Results indicated interesting differences between lower secondary education, upper secondary education and vocational education and training teachers. The effect size values (*r*_U_ statistic) ranged between 0.43 and 0.63, highlighting the significant effect of teachers’ teaching experience on the six teaching skills domains: learning climate, efficient classroom management, clarity of instruction, activating teaching, differentiation, and teaching learning strategies. Those teachers with less teaching experience were the ones who showed higher scores. Findings from the regression model showed that educational level had a significant predictive effect on the six teaching skills domains, mainly for male teachers. However, in several domains female teachers were perceived by students to outperform their male counterparts.

## Introduction

Teaching behavior is an important element in supporting student learning and achievement, along with student ability and background. The effect of teaching seems to be the result of a complex interplay between personal and environmental factors requiring deep analysis for a more thorough understanding on the integration and effect of each of these two factors (Kyriakides and Creemers, 2009; Kyriakides et al., 2009; Sammons and Bakkum, 2011).

In many cases, teachers’ initial training is not based on previous experiences of effective teaching practices so when prospective teachers finish their training they usually feel the need for guidance (Maulana et al., 2015b,c). Besides, learning to teach is best accomplished with assistance or support (Tschannen-Moran and Woolfolk, 2001; Conway and Clark, 2003). Similarly, experienced teachers also need guidance to maintain and develop their knowledge and skills, to reflect and collaborate with colleagues (Panayiotis et al., 2011; Kini and Podolsky, 2016). In short, identifying teachers’ weak points may help them improve their teaching competence making them more effective. Research has shown that teacher behavior predicts student learning, cognitive, and affective outcomes (De Jong and Westerhof, 2001; Opdenakker and Van Damme, 2006; Sammons and Bakkum, 2011; Van de Grift et al., 2014; Maulana et al., 2015b,c; Van der Lans et al., 2015). However, there is an important and ongoing need to identify potential explanatory variables contributing to the quality of teaching behavior. Therefore, teacher evaluation may offer the opportunity to resolve the missing link between teacher learning process and teacher practice, giving teachers the opportunity to progress (Tuytens and Devos, 2014).

In Spain, teachers in higher education are used to having their teaching evaluated using different procedures. However, in other educational levels there is a lack of systematic procedures for evaluation. Only two Spanish Autonomous Communities have developed official systems for teacher evaluation. Since 2007, in the autonomous community of Asturias there has been a *Plan de Evaluación Docente* [Teachers’ Assessment Plan], which provides economic incentives for those teachers with a positive evaluation. This plan was created with the idea that teaching assessment should be based on objective results, considering student outcomes along with procedural and contextual elements which make it possible to give feedback during teaching and learning.

In Catalonia, teachers’ effectiveness in attaining planned objectives and the improvement in teaching results are also evaluated. Since 2016, teachers have been able to voluntarily apply for an individual evaluation which includes aspects such as pedagogical competence, use of teaching techniques, and participation in the school (Bolívar et al., 2016). Despite the current move toward teacher evaluation in Spain, scientific research into teaching behavior in this country remains scarce. In particular, little is known about: (1) the general level of teachers’ teaching behavior quality, and (2) the influence of various contextual and personal characteristics in explaining differences in teachers’ teaching behavior. This knowledge would be useful to guide current and future professional development programs for teachers.

The lack of systematic research about teaching behavior in Spain, and the importance to test whether certain personal and contextual variables matter for teaching behavior, are the motivation for this study. Based on current results of a popular international testing studies such as the Program for International Student Assessment (PISA), student performance in Spain (*M* = 486) was below the Organization for Economic Cooperation and Development (*M* = 500) average (OECD, 2018). Research on teaching effectiveness has indicated that about 15–25% of differences in students’ achievement can be explained by the work of teachers (e.g., Bosker and Witziers, 1996; Aaronson et al., 2007; Houtveen and Van de Grift, 2007). Hence, teaching quality matters for explaining differences in student performance. We expect that the rather low performance of students in the international testing study in Spain will be reflected in the general quality of teachers.

## Effective Teaching Behavior

Van de Grift (2007) has developed a teaching behavior framework based on research into teaching and teacher effectiveness. The model consists of six teaching domains: safe learning climate, efficient classroom management, clarity of instruction, activating teaching, differentiation, and teaching learning strategies (Van de Grift et al., 2014; Maulana et al., 2015b; Van der Lans et al., 2015). These six teaching behavior domains are recognized as highly important teaching indicators for student learning and outcomes (see Van de Grift, 2007; Maulana et al., 2015a).

Furthermore, Van de Grift’s model is very useful as it gives us an idea of the observable teaching behaviors exhibited by teachers in their classrooms. While some of these behaviors are easily acquired and deployed by most teachers, others are more complex and many teachers may find it difficult to employ them in their teaching (Van de Grift et al., 2014; Maulana et al., 2015a). The less complex domains are safe learning climate, efficient classroom management, and clarity of instruction. Activating teaching, teaching learning strategies, and differentiation appear to be more difficult for teachers and need further attention to master (Van de Grift et al., 2014; Van der Lans et al., 2015).

## Contextual and Personal Factors Influencing Teaching Behavior

Previous research has indicated that various personal characteristics, such as teaching experience and gender, explain differences in teaching behavior and student outcomes. Antoniou et al. (2011) found that the amount of a teacher’s experience had a positive effect on student achievement. Staiger and Rockoff (2010) concluded that teacher effectiveness improved rapidly in the 1st year or two of their teaching careers and leveled off rapidly in subsequent years. Based on a review of 30 American studies, Kini and Podolsky (2016) found that although the gains from experience were highest in teachers’ first few years, the gains continued during the second and often third decades of their careers. Their research suggested that teaching experience was positively associated with student outcomes. Opdenakker et al. (2012) found in their study in the Netherlands that teacher behavior (in terms of teacher–student interpersonal relationship: teacher proximity and teacher influence) tended to decrease during the school year. As the literature has indicated that high quality teacher–student interpersonal relationships corresponded to better student outcomes, this finding pointed toward a deterioration of the (current Dutch) learning environment.

In contrast to other studies, Conway and Clark (2003), in their study with pre-service teachers, found that class management tasks were not as important as initially thought given their status as novice teachers. There are also some studies which have shown that teacher efficacy and student guidance depended on the amount of teaching experience, and that experienced teachers had higher scores compared to novice teachers (Van Petegem et al., 2005). In a widely read comprehensive review of teaching effectiveness, Hattie (2003) found a difference in effectiveness between teachers with different teaching experience as regards the way they represented their classrooms, the degree of challenges they offered students and the depth of processing attained by the students. Hattie found that students who were taught by experienced teachers exhibited a better understanding of the concepts, more coherent integration and a higher level of abstraction than other students taught by less experienced teachers. Similarly, other studies (e.g., Maulana et al., 2012, 2013) also obtained significant differences suggesting that experienced teachers were better than inexperienced teachers in terms of teacher involvement, teacher friendliness, and reviewing lessons to ensure mastery learning.

Furthermore, several studies with Dutch and Indonesian teachers indicated that teacher gender was also an important predictor of teacher classroom management, concluding that male teachers tended to maintain order better than their female colleagues and that friendliness was perceived to be lower in female teachers classes compared to male teachers (Opdenakker and Van Damme, 2007; Opdenakker et al., 2012). The studies suggested that female teachers seemed to be stricter, less cooperative and less friendly than male teachers. Van Petegem et al. (2005), looking at teachers’ submitting – opposing interpersonal behavior, also concluded that male teachers were more dissatisfied and uncertain compared to their female colleagues and that compared to colleagues with job security male teachers without job security perceived themselves more as leaders with helpful and friendly interpersonal behavior. In another study female preservice teachers displayed better teaching behavior and clarity of instruction compared to male preservice teachers (Maulana et al., 2017). Additionally, Maulana et al. (2012) found that female teachers appeared to spend less time on student work but more time on closing the lessons compared to their male colleagues. In short, the role of teacher gender on teaching behavior seemed not to be straightforward, depending on the context of the study and the measures used to capture teacher behavior.

Other research has focused on the analysis of certain educational levels covering primary (e.g., Van de Grift, 2007, 2014; Kyriakides and Creemers, 2009; Kyriakides et al., 2009; Antoniou et al., 2011; Boonen et al., 2014) and secondary education (e.g., De Jong and Westerhof, 2001; Den Brok et al., 2004; Opdenakker and Van Damme, 2006, 2007; Opdenakker et al., 2012; Tuytens and Devos, 2014; Van de Grift et al., 2014; Maulana et al., 2015b,c; Van der Lans et al., 2015, 2016) but there is a lack of research focusing on the comparison of teachers’ effective behavior in different types of secondary education except for research by Opdenakker and Van Damme (2006) analyzing differences between various types of secondary schools in Flanders. They concluded that ASO schools (which offer academic/general track) and multitrack schools performed significantly better than autonomous middle schools and TSO/TBO schools (which only offer the technical and vocational tracks from the 3rd year of secondary education and above). It was also found that in TSO/BSO schools, students had significantly worse achievement in mathematics compared to ASO schools, autonomous middle schools or multitrack schools.

In Spain, the study of differences between different kinds of schools seems crucial as the 1990 reform of the Spanish Education Act (Organic Law for the General Organization of the Educational System) established the integration of vocational and academic streams in the same schools, approved compulsory education for students from 6 to 16 years old and gave vocational education and training a more prestigious status. All these changes have encouraged teachers to adapt their instructional strategies in order to deal with the new profile of students and programs.

## Student Perceptions of Teachers’ Teaching Behavior

The use of student evaluation of teaching (SET) makes it possible to create a picture of teaching behavior built from a representative sample of day to day teachers’ practices (Hoyt and Pallet, 1999; De Jong and Westerhof, 2001). Additionally, there is evidence that student perceptions are more predictive of student learning outcomes than other methods such as external observations or teachers’ subjective perceptions of their own teaching behavior (Maulana et al., 2015b). Although it is a more cost effective method, the reliability and validity of student ratings is, to some extent, compromised because student evaluations usually reflect their expectations about the teacher (Mateo, 2000; De Jong and Westerhof, 2001; Maulana et al., 2015b,c; Van der Lans et al., 2015). The connection between the validity of SET and student age is not clear-cut. However, Den Brok et al. (2004) argued that secondary students were able to provide ratings of teacher behavior that were sufficiently stable, reliable, valid, and predictive for teaching evaluation research. On the other hand, Van de Grift (2007) and Mateo (2000) expressed their doubts about the capability of very young children to make objective, stable assessments. Considering Martínez Rizo (2012) conclusions the validity of student ratings may depend on the content of the measures: they may not be accurate for assessing teachers’ knowledge or planning strategies, but could be useful when the focus is on the teaching strategies used in class, the content of a subject or their teaching effectiveness.

This study examined student perceptions of teachers’ teaching behavior and the role of certain background characteristics (teaching experience, gender, and educational level) in explaining differences in perceived teaching behavior. To guide the study the following research questions were formulated:

(1) What is the general level of secondary school teachers’ teaching behavior as perceived by their students in Spain?
(2) How do background variables (student educational level, teacher teaching experience, and teacher gender) explain differences in student perceptions of teaching behavior?

Following these research questions, two hypotheses were set up. Based on the previous empirical evidence regarding the rather low performance of Spanish students in the international study (OECD, 2018), the strong importance of teaching quality for student achievement (e.g., Bosker and Witziers, 1996; Aaronson et al., 2007; Houtveen and Van de Grift, 2007), and the complexity level of teaching behavior domains (Van de Grift et al., 2014; Maulana et al., 2015a), we hypothesize that the perceived general level of secondary school teachers’ teaching behavior will be sufficient. Particularly, the sufficient quality level will be more evident for more basic teaching skills (learning climate, efficient classroom management, clarity of instruction) compared with more complex teaching skills (activating teaching, differentiation, and teaching learning strategies). The expectation regarding the role of teacher characteristics and teaching behavior is less clear-cut due to the absence of such studies in Spain. By connecting to the relevant literature from different national settings (e.g., Antoniou et al., 2011; Opdenakker et al., 2012; Maulana et al., 2017), we expect that educational level, teaching experience and teacher gender will explain differences in perceived teaching behavior to some extent.

## Materials and Methods

### Participants

The participants were 7,114 students taught by 410 teachers attending 56 public and private schools in Spain. A total of 3,577 of the sample were boys (51%) and 3,415 were girls (49%). A total of 122 students did not report their gender. 2,970 (41.9%) students had male teachers and 4,122 (58.1%) female ones. Just under three quarters of the students (*n* = 5,112; 71.9%) were in lower secondary education (this secondary educational level comprises 4 years and is aimed at students aged 12–16 years), 1,105 students (15.5%) were in upper secondary education (last 2 years of secondary school, students with 16–18 years old) and 897 students (12.6%) were in vocational education and training (1 or 2 years, for students with more than 16 years old). A total of 3,183 students (44.7%) were at academic schools, 205 (2.9%) at vocational schools and 3,726 (52.4%) at schools which had academic and vocational programs simultaneously (multitrack). A total of 4,702 students (66.1%) were from public schools whereas 2,412 (33.9%) were from private schools. Teachers were classified into four categories according to their teaching experience: less than 10 years = less experienced (*M* = 6 years); between 11 and 20 years of experience = moderately experienced (*M* = 16 years); between 21 and 30 years of experience = very experienced teachers (*M* = 26 years) and teachers with more than 30 years of teaching experience = extremely experienced (*M* = 35 years).

The initial intention of the research team was to use the probability proportional to size sampling technique. However, due to reticence found in most of the schools a non-probabilistic convenience sampling method had to be used.

### Instruments

#### Teaching Behavior

To gather student perceptions of teachers’ behavior, we used the My Teacher questionnaire developed by Maulana et al. (2015b). The questionnaire was translated and back-translated for use in the Spanish context following the guidelines provided by Hambleton et al. (2005). Two researchers with fluent English and deep knowledge of the Spanish education system performed the initial Spanish translation. Subsequently, a university research panel assessed the translation results focusing on the item level to make sure that the content of each item was representative of the Spanish education system. Additionally, they gave opinions about the appropriate content and structure for use in the Spanish secondary education level. The initial Spanish translation was then translated back into English. The Spanish version and the back translated English version were checked by the second university research panel including the original developer of the questionnaire and a university professor of Spanish language.

The questionnaire consisted of 41 items measuring six domains including safe learning climate (α = 0.66), efficient classroom management (α = 0.76), clarity of instruction (α = 0.70), activating teaching (α = 0.80), differentiation (α = 0.60), and teaching learning strategies (α = 0.71) (Inda-Caro et al., 2018). Item response was provided using a rating-scale ranging from 1 (completely untrue) to 4 (completely true). Teachers were asked about their professional experience as teachers and categorical responses were used to gather information about teaching experience (0 = less than 10 years, 1 = between 11 and 20 years, 2 = between 21 and 30 years, 4 = more than 30 years), gender (0 = male, 1 = female) and educational level (0 = lower secondary education, 1 = upper secondary education, 2 = vocational education and training).

#### Academic Engagement Scale

A 10 item scale in which students rated their behavioral (five items) and emotional (five items) engagement was used. This measure was based on the engagement scale of Skinner et al. (2008). All items were provided on a 4 point rating scale, ranging from 1 (completely untrue) to 4 (completely true). In the Spanish version, the Cronbach’s alpha value was 0.88 (Inda-Caro et al., 2018). Behavioral engagement was assessed by items such as: “In this class I try hard to do well” or “In this class I participate in class discussions.” Examples to assess emotional engagement were items such as: “In this class I feel good” or “In this class I enjoy learning new things.”

Table 1 shows the instrument validity in terms of mean inter-scale correlations and predictive validity, showing the correlation between the six domains and the two criterion constructs: students’ behavioral and emotional engagement.

**Table 1:** Correlations between teachers’ teaching behavior domains and students’ academic engagement.

	LC	EM	CI	AT	DI	TL	BE	EE
Learning climate (LC)								
Efficient classroom management (EM)	0.59^∗∗^							
Clarity of instruction (CI)	0.49^∗∗^	0.61^∗∗^						
Activating teaching (AT)	0.51^∗∗^	0.54^∗∗^	0.54^∗∗^					
Differentiation (DI)	0.41^∗∗^	0.48^∗∗^	0.50^∗∗^	0.55^∗∗^				
Teaching learning strategies (TL)	0.36^∗∗^	0.38^∗∗^	0.40^∗∗^	0.51^∗∗^	0.48^∗∗^			
Behavioral engagement (BE)	0.18^∗∗^	0.19^∗∗^	0.17^∗∗^	0.22^∗∗^	0.19^∗∗^	0.17^∗∗^		
Emotional engagement (EE)	0.25^∗∗^	0.25^∗∗^	0.24^∗∗^	0.31^∗∗^	0.26^∗∗^	0.27^∗∗^	0.49^∗∗^	
*M*	3.15	3.11	3.12	2.99	2.96	2.77	3.09	3.13
*SD*	0.50	0.48	0.51	0.53	0.60	0.61	0.56	0.62


*^∗^p < 0.05, ^∗∗^p < 0.01, ^∗∗∗^p < 0.001.*

### Procedure

In the spring term of the school year, the members of the research group requested student participation and collected data at each school. After a brief presentation in which the researchers described the purpose of the study, the students were asked to fill out the questionnaire which took about 30 min to complete. The questionnaires were completed in normal class time. There was no remuneration or course credit for participation and anonymity was guaranteed. The participation in the research was voluntary and no parents withheld their consent.

Although a full review and approval of the research is not required according to Spanish local and national regulations, this study was carried out in accordance with the recommendations of the Institutional Review Board from all the schools (approved by the head teachers and also in the *Comisión de Coordinación Pedagógica* and *Claustro*). The study also received the approval of the Department of Education of the Principality of Asturias (Spain) which selected and published which projects could be developed in any of the schools of their competence. At the beginning of the academic year, all schools asked families for a written and informed consent so that their children were given permission to participate in any project or research of the school. Moreover, ICALT3 -name of the general project- was included in the catalog of authorized research (Type C. New research and innovation projects of the University of Oviedo which involve cooperation with schools).

### Data Analysis

To answer the first research question, we carried out descriptive analyses. Following Maulana et al. (2015a), the classification metric of the scales was interpreted as follows: 1.00–1.99 (insufficient), 2.00–2.99 (sufficient), and 3.00–4.00 (good). To answer the second research question, we performed non-parametric tests. Mann–Whitney *U* test was used for examining the six teaching domains and teachers’ gender. Kruskal–Wallis non-parametric test was used for the analysis of teachers’ teaching experience. Afterwards, in order to analyze differences among teachers’ teaching experience, a *post hoc* analysis with Mann–Whitney *U* test and Bonferroni correction was performed. Non-parametric tests were used instead of parametric tests, since the criterion variables (teaching behavior domains) did not follow a normal distribution as indicated by the Kolmogorov–Smirnov test (*p* < 0.001) and kurtosis values (higher than 1).

Effect sizes (ES) were calculated through *r*_U_ for Mann–Whitney *U* test, considering a *r*_U_ value higher than 0.50 as very good, between 0.10 and 0.30 medium and 0.10 or less as small effect size. The value of *r*_U_ was calculated by dividing _U_ among the possible comparisons with the units from both samples (*n*1, *n*2), with _U_ representing the frequency of having more probability of being in one group than in another group (Grissom and Kim, 2012). In addition, a linear regression model was applied to determine the importance of the three background variables for each teaching behavior domain. Possible interaction effects between variables were also examined.

## Results

### General Level of Secondary School Teachers’ Teaching Behavior in Spain

Following the metric criteria as proposed by Maulana et al. (2015a), we found that in general, students perceived teachers’ learning climate (*M* = 3.15, *SD* = 0.50), efficient classroom management (*M* = 3.11, *SD* = 0.48) and instructional clarity (*M* = 3.12, *SD* = 0.51) as good, while they perceived teachers’ activating teaching (*M* = 2.99, *SD* = 0.53), differentiation (*M* = 2.96, *SD* = 0.60), and teaching learning strategies (*M* = 2.77, *SD* = 0.61) as sufficient.

### Teaching Experience, Gender, Educational Level and Teaching Behavior

#### Lower Secondary Education Teachers

**FIGURE 1:**
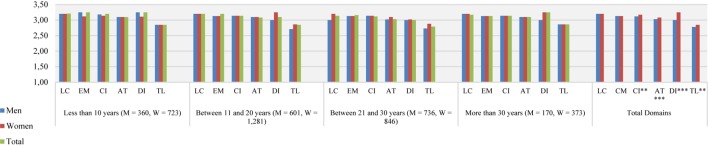
Distribution of teaching behavior domains by gender and teaching experience. Lower secondary education. Total *n* = 5,090, Men *n* = 1,867, Women *n* = 3,223. M, men; W, women. LC, learning climate; EM, efficient classroom management; CI, clarity of instruction; AT, activating teaching; DI, differentiation; TL, teaching learning strategies. ^∗^*p* < 0.05, ^∗∗^*p* < 0.01, ^∗∗∗^*p* < 0.001.

Figure 1 shows that female teachers in lower secondary education were rated higher than male teachers in four domains: instructional clarity (*U* = 2,881,773.00, *p* < 0.01, *r*_U_ = 0.48), activating teaching (*U* = 2,852,936.50, *p* < 0.001, *r*_U_ = 0.47), differentiation (*U* = 2,816,086.50, *p* < 0.001, *r*_U_ = 0.47) and teaching learning strategies (*U* = 2,874,244.50, *p* < 0.01, *r*_U_ = 0.48).

We found significant differences in all domains by teaching experience (Table 2). With the *post hoc* tests, the differences were found for less experienced teachers (teachers with less than 10 years of experience) compared to very experienced teachers (those with between 21 and 30 years of experience). Less experienced teachers were perceived as having better performance in learning climate, efficient classroom management, instructional clarity, differentiation and teaching learning strategies compared to their more experienced colleagues. Similar differences were visible in efficient classroom management and instructional clarity in comparison with extremely experienced teachers (those with more than 30 years of experience). Moderately experienced teachers (teachers with between 11 and 20 years of experience) were also rated higher in efficient classroom management and activating teaching compared to very experienced teachers.

**Table 2:** *Post hoc* analysis of domains considering teaching experience in lower secondary education teachers.

	≤10 years	11–20 years	21–30 years
≤10 years			
11–20 years			
21–30 years	Learning climate, *U* = 783,894.5^∗∗∗^, *r*_u_ = 0.46		
	Efficient classroom management, *U* = 763,119.5^∗∗∗^, *r*_u_ = 0.46	Efficient classroom management, *U* = 1,397.495^∗∗^, *r*_u_ = 0.47	
	Instructional clarity, *U* = 778,408^∗∗∗^, *r*_u_ = 0.45	Activating teaching, *U* = 1,397.245^∗∗^, *r*_u_ = 0.47	
	Differentiation, *U* = 794,098.5^∗∗^, *r*_u_ = 0.46		
	Teaching learning strategies, *U* = 795,670.5^∗∗^, *r*_u_ = 0.46		
≥30 years	Efficient classroom management, *U* = 271,045.50^∗^, *r*_u_ = 0.46		
	Instructional clarity, *U* = 263,563^∗∗^, *r*_u_ = 0.45		


*Learning climate χ^*2*^ = 15.07^∗∗^; Efficient classroom management χ^*2*^ = 24.87^∗∗∗^; Instructional clarity χ^*2*^ = 21.28^∗∗∗^; Activating teaching χ^*2*^ = 10.10^∗^; Differentiation χ^*2*^ = 12.26^∗∗^; Teaching learning strategies χ^*2*^ = 12.71^∗∗^. ^∗^p < 0.05, ^∗∗^p < 0.01, ^∗∗∗^p < 0.001*.

#### Upper Secondary Education Teachers

**FIGURE 2:**
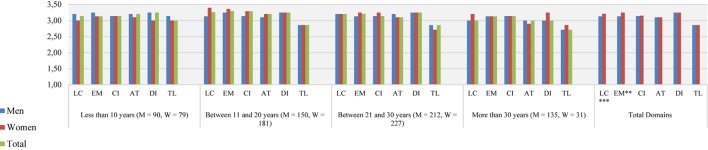
Distribution of teaching domains by gender and teaching experience. Upper secondary education. Total *n* = 1,105, Men *n* = 587, Women *n* = 518. M, men; W, women; LC, learning climate; EM, efficient classroom management; CI, clarity of instruction; AT, activating teaching; DI, differentiation; TL, teaching learning strategies. ^∗^*p* < 0.05, ^∗∗^*p* < 0.01, ^∗∗∗^*p* < 0.001.

Figure 2 shows that students perceived female teachers in upper secondary education to have better learning climates than their male colleagues (*U* = 135,379.50, *p* < 0.001; *r*_U_ = 0.45) and that female teachers were perceived to manage the classroom better than male teachers (*U* = 137,293.50, *p* < 0.01, *r*_U_ = 0.45).

In terms of professional teaching experience, *post hoc* tests results showed that main differences were found between less experienced teachers and more experienced teachers (see Table 3). Less experienced teachers had higher ratings in teaching learning strategies compared to very experienced teachers. They were also rated higher than more experienced teachers in learning climate, activating teaching, and teaching learning strategies. However, less experienced teachers had lower scores than moderately experienced teachers in learning climate and efficient classroom management.

**Table 3:** *Post hoc* analysis of domains considering teaching experience in upper secondary education teachers.

	≤10 years	11–20 years	21–30 years
≤10 years			
11–20 years	Learning climate, *U* = 23,557^∗^, *r*_u_ = 0.60		
	Efficient classroom management; *U* = 22,972^∗∗^, *r*_u_ = 0.59		
21–30 years	Teaching learning strategies, *U* = 29,532.5^∗∗∗^, *r*_u_ = 0.40	Efficient classroom management, *U* = 63,386.50^∗∗^, *r*_u_ = 0.63	
≥30 years	Learning climate, *U* = 11,541^∗^, *r*_u_ = 0.41	Learning climate, *U* = 18,928^∗∗∗^, *r*_u_ = 0.49 Efficient classroom management, *U* = 19,958^∗∗∗^, *r*_u_ = 0.52	Learning climate, *U* = 28,220^∗∗∗^, *r*_u_ = 0.39
	Activating teaching, *U* = 11,404.5^∗∗^, *r*_u_ = 0.41	Instructional clarity, *U* = 23,341.50^∗^, *r*_u_ = 0.61 Activating teaching, *U* = 22,091^∗∗∗^, *r*_u_ = 0.58	Efficient classroom management, *U* = 31,070^∗^, *r*_u_ = 0.43
	Teaching learning strategies, *U* = 10,096.50^∗∗∗^, *r*_u_ = 0.36	Differentiation, *U* = 22,703^∗∗^, *r*_u_ = 0.59 Teaching learning strategies, *U* = 22,847.50^∗∗^, *r*_u_ = 0.60	


*Learning climate χ^*2*^ = 35.91^∗∗∗^; Efficient classroom management χ^*2*^ = 28.39^∗∗∗^; Instructional clarity χ^*2*^ = 9.06^∗^; Activating teaching χ^*2*^ = 14.51^∗∗^; Differentiation χ^*2*^ = 10.54^∗∗^; Teaching learning strategies χ^*2*^ = 24.92^∗∗∗^. ^∗^p < 0.05, ^∗∗^p < 0.01, ^∗∗∗^p < 0.001*.

Furthermore, moderately experienced teachers were scored more highly than very experienced teachers in efficient classroom management by their students (see Figure 2 and Table 3). They also had better ratings from their students than extremely experienced teachers in the six effective teaching domains. Finally, very experienced teachers demonstrated better quality in learning climate and efficient classroom management than extremely experienced teachers.

#### Vocational Education and Training Teachers

**FIGURE 3:**
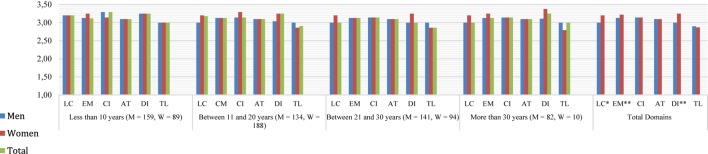
Distribution of teaching behavior domains by gender and teaching experience. Vocational education and training teachers. Total *n* = 897, Men *n* = 516, Women *n* = 381. M, men; W, women. LC, learning climate; EM, efficient classroom management; CI, clarity of instruction; AT, activating teaching; DI, differentiation; TL, teaching learning strategies. ^∗^*p* < 0.05, ^∗∗^*p* < 0.01, ^∗∗∗^*p* < 0.001.

Figure 3 shows that female teachers in vocational education and training had better ratings in learning climate (*U* = 90,708.50, *p* < 0.05; *r*_U_ = 0.46), efficient classroom management (*U* = 86,587.50, *p* < 0.01; *r*_U_ = 0.44) and differentiation (*U* = 88,963, *p* < 0.01; *r*_U_ = 0.45). Results of *post hoc* tests showed that teaching experience was only a differential factor in learning climate (χ^2^ = 35.91, *p* < 0.05), specifically, in less experienced teachers and very experienced teachers (*U* = 24,970.50, *p* < 0.05, *r*_U_ = 0.43).

### Personal and Contextual Factors

**FIGURE 4:**
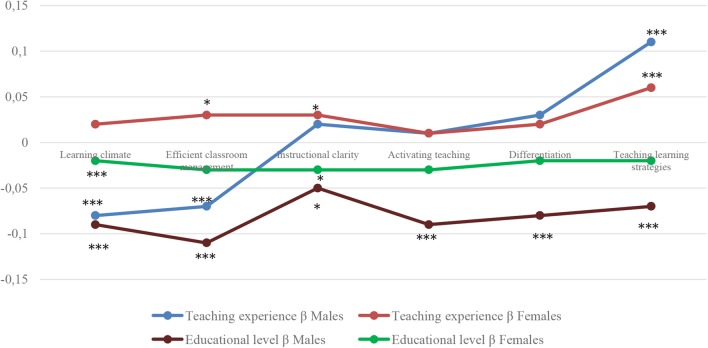
Predictive values of teacher teaching experience and education level for male and female teachers for the six teaching behavior domains. *R*^2^ Females: Learning climate = 0.1%, Efficient classroom management = 0.2%, Instructional clarity = 0.2%, Activating teaching = 0.1%, Differentiation = 0.1% and Teaching learning strategies = 0.4%. *R*^2^ Males: Learning climate = 2%, Efficient classroom management = 2%, Instructional clarity = 0.3%, Activating teaching = 1%, Differentiation = 1% and Teaching learning strategies = 2%.^∗^*p* < 0.05, ^∗∗^*p* < 0.01, ^∗∗∗^*p* < 0.001.

The regression model results (Figure 4) showed a statistically significant effect of teaching experience and educational level on the learning climate domain for male teachers, but not for female ones. Results suggested that students perceived male teachers with less teaching experience and those working in lower secondary education having better learning climates than more experienced, upper secondary education or vocational education and training male teachers. For efficient classroom management, only teaching experience appeared to have a statistically significant effect for both male (negative effect) and female (positive effect) teachers. Results revealed that when male teachers had more teaching experience, their student ratings in efficient classroom management tended to decrease, whereas for female teachers, professional experience had a positive impact on their classroom management. The educational level had a negative influence on efficient classroom management only for male teachers: in lower secondary education they had better ratings than their colleagues in the other two levels.

Student perceptions of instructional clarity seemed to be predicted by the educational level for both male and female teachers. The results showed that lower secondary education teachers had better ratings than upper secondary education or vocational education and training teachers. Nevertheless, teaching experience only predicted instructional clarity for female teachers. Besides, female teachers with more teaching experience were seen as more capable of organizing their instruction clearly. Our results also showed that the educational level had a statistically significant negative effect in activating teaching and differentiation but only for male teachers. This inverse relationship implied that male teachers in lower secondary education were perceived to outperform their male colleagues in upper secondary education and vocational education and training. Teaching experience did not predict ratings in activating teaching or differentiation neither for male teachers nor for female ones. Finally, teaching experience had a positive effect on teaching learning strategies for both male and female teachers. Unlike the other domains, male and female teachers with more professional teaching experience showed better ratings in this last domain. On the other hand, the educational level only had a negative influence for male teachers: the ability to cope with teaching learning strategies seemed to be perceived better for lower secondary education teachers. It is necessary to note that *R*^2^ values were not high (lower than 0.30). Consequently, these data suggested the need to deepen the investigation in which other factors might be influencing the six teaching skills domains.

The interaction effect between teaching experience, education level, and teacher gender was also analyzed. The interaction between gender × education level had significant effects in instructional clarity [*F*(2, 7,068) = 3.15; *p* < 0.05; η^2^ = 0.01]. Considering the interaction between gender × teaching experience, significant effects were observed in: learning climate [*F*(3, 7,068) = 7.72; *p* < 0.001; η^2^ = 0.003], efficient classroom management [*F*(3, 7,068) = 8.45; *p* < 0.001; η^2^ = 0.004], instructional clarity [*F*(3, 7,068) = 7.35; *p* < 0.001; η^2^ = 0.003], activating teaching [*F*(3, 7,068) = 5.52; *p* < 0.001; η^2^ = 0.002], differentiation [*F*(3, 7,068) = 2.92; *p* < 0.05; η^2^ = 0.001] and teaching learning strategies [*F*(3, 7,068) = 3.56; *p* < 0.01; η^2^ = 0.002]. The interaction between educational level × teaching experience had significant effects for learning climate [*F*(6, 7,068) = 6.33; *p* < 0.001; η^2^ = 0.005], efficient classroom management [*F*(6, 7,068) = 4.00; *p* < 0.001; η^2^ = 0.003], instructional clarity [*F*(6, 7,068) = 3.15; *p* < 0.01; η^2^ = 0.003], and activating teaching [*F*(6, 7,068) = 2.59; *p* < 0.01; η^2^ = 0.002]. The effect of the interaction among the three predictive variables (gender × education level × teaching experience) was significant for learning climate [*F*(6, 7,068) = 5.11; *p* < 0.001; η^2^ = 0.004], efficient classroom management [*F*(6, 7,068) = 6.42; *p* < 0.001; η^2^ = 0.005], and instructional clarity [*F*(3, 7,068) = 2.21; *p* < 0.05; η^2^ = 0.002].

Following the previous findings regarding general interactions, the interactions by gender were considered separately. For male teachers, the findings revealed that the interaction between teaching experience x educational level had significant effects for learning climate [*F*(6, 2,958) = 4.97; *p* < 0.001; η^2^ = 0.01], efficient classroom management [*F*(6, 2,958) = 2.31; *p* < 0.05; η^2^ = 0.01]; activating teaching [*F*(6, 2,958) = 2.19; *p* < 0.05; η^2^ = 0.004] and teaching learning strategies [*F*(6, 2,958) = 2.74; *p* < 0.01; η^2^ = 0.01]. Regarding female teachers, the significant interaction effects were found for learning climate [*F*(6, 4,110) = 7.67; *p* < 0.001; η^2^ = 0.01]; efficient classroom management [*F*(6, 4,110) = 8.46; *p* < 0.001; η^2^ = 0.01]; instructional clarity [*F*(6, 4,110) = 3.67; *p* < 0.001; η^2^ = 0.01]; and activating teaching [*F*(6, 4,110) = 2.35; *p* < 0.05; η^2^ = 0.003].

## Discussion

This study examined the general level of effective teaching behavior of secondary education teachers as perceived by their students in Spain. Our findings showed substantial differences in the level of perceived effective teaching behavior found in each domain and confirmed the role of teaching experience, gender, and educational level to explain differences in perceived teaching behavior.

Our findings partially confirmed our first hypothesis showing that the perceived general level of secondary school teachers’ teaching behavior varied from sufficient to good depending on the teaching behavior domains (hypothesis 1). Consistent with previous research (e.g., Van de Grift et al., 2014; Maulana et al., 2017), our results confirmed that in Spain, teachers in general were perceived to exhibit lower quality teaching behavior in the more complex teaching domains (i.e., activating teaching, differentiation and teaching learning strategies), compared to the less complex domains. Kyriakides et al. (2009) found that teachers who used more advanced types of teaching behavior were more effective (i.e., producing higher performing students) than those using relatively easy behaviors. The fact that perceived teaching behavior in the more complex domains were not at the good level might partially explain the rather low performance of students in the international testing studies in Spain (cf. OECD, 2018). Hence, efforts should be made to improve teachers’ skills in these more complex domains. This finding gives clues about the necessity to base teachers’ training on those more challenging domains as they have been identified as weak points which may help improve teachers’ competence, without paying less attention to more basic teaching skills. Moreover, the strong connection between effective teaching research and teachers training programs may help improve the core focus of teachers’ training with the aim to obtain better outcomes with the students.

In line with our expectation, we found that educational level, teaching experience, and teacher gender could explain differences in perceived teaching behavior (hypothesis 2). In contrast to some previous studies (Maulana et al., 2015b,c, 2017), our study showed that it is not the most inexperienced teachers who seem to need more support in the improvement of teaching behavior, but rather those groups with more teaching experience. Teachers with less experience had the highest scores in most domains including learning climate, efficient classroom management, and teaching learning strategies. This pattern was particularly visible in lower and upper secondary education. One possible explanation for this finding could be related to the openness to affective needs of students (Kees and Lashwood, 1996). In line with this demand, students might prefer younger teachers as they can relate more to their reality or even they might consider that young teachers fulfill more adequately the affective needs they experience during adolescence. Further research is needed to explore this perspective in the analysis of teacher – student interactions. Another possible explanation could be that in 2010 Spain started a significant change in the initial training required to be a secondary education or vocational education and training teacher: apart from holding a relevant 4-year University Degree, prospective teachers have now to do a Master’s degree in which they are taught full time about pedagogical, psychological and didactic issues. This change also reinforced the need for continuous professional development and to connect teachers’ needs with this development, giving teachers formative feedback about their teaching (Van der Lans et al., 2015). Tuytens and Devos (2014) reported that the amount of experience was negatively related to the perceived utility of feedback after the evaluation process, so teachers who are more experienced seem to be a target group who need very clear and concise feedback. To date, Spain has not established a support system to give teachers this information about the domains they should improve or to help them make their teaching activities more effective. Such a system would be desirable as it may help teachers highlight what they need to learn in order to become more effective.

Our finding is in line with the research of De Jager et al. (2017) showing that teachers with less than 15 years of teaching experiences displayed more effective teaching behavior compared with more experienced teachers, from the student’s point of view. They found that the most experienced teachers (more than 30 years of teaching experience) displayed the least effective teaching behavior. They argued that although most experienced teachers acquired more content knowledge from experience, they might lack the motivation to adapt to changes of the more contemporary curriculum (De Jager et al., 2017). This reasoning might apply to the context of Spain as well. Nevertheless, more research is needed to validate this speculation.

Some studies have indicated that the association between teaching experience and teaching quality was generally positive, but the relational pattern seemed to be non-linear. The largest improvement in teaching quality was found to be related to the first few years of teaching, and the improvement seemed to be less visible after about 5 years of experience (Rivkin et al., 2005; Rice, 2013). Similarly, research showed that a slight decline in teaching quality was visible for teachers with more than 15 years of experience, with less experienced teacher showing better levels of complex skills (i.e., differentiated instruction) compared with more experienced teachers (De Jager et al., 2017). Those research findings are consistent with findings of the current study to some extent. Furthermore, the relation between experience and teaching quality as perceived by students might also be affected by another teacher characteristics such as self-efficacy. For example, Klassen and Chiu (2010) found that teachers’ self-efficacy in classroom management increased from early career/mid- career, but deteriorated subsequently. Future research should incorporate the role of self-efficacy in explaining the interplay between teaching experience and teaching quality further.

Female teachers were perceived as having higher quality in almost all domains of teaching behavior. These results were somewhat similar to results from Maulana et al. (2017) who found that female preservice teachers exhibited better classroom management and clarity of instruction compared to male preservice teachers. In our study, female lower secondary education teachers were rated higher than their male colleagues in instructional clarity, activating teaching, differentiation, and teaching learning strategies (ES = 0.47–0.48, medium effect). For male upper secondary education teachers, these significant differences were only apparent in learning climate and efficient classroom management (ES = 0.45, medium effect). Finally, female vocational education and training teachers were rated higher in learning climate, efficient classroom management, and differentiation (ES = 0.44–0.46, medium effect). These results in favor of female teachers may be related to a traditional view of teaching, which is linked to an idea of care and helping others or as a feminine profession. Furthermore, students may perceive that their female teachers’ activities are kinder or fit better with their preconceived idea of a good or favorable teacher. The Spanish government has recently undertaken several measures in pursuit of gender equality, so teachers and teacher education may be considered as one of the priorities of these initiatives. However, these differences should be considered with prudence, since the gender effect size, although at the high end of medium level, was not large.

Vocational education and training teachers should be investigated more deeply, as according to students’ opinion, they exhibited better strategies and more innovative teaching practices. This may be related to the fact that it constitutes a more practical training, to student profiles, to the lower pressure experienced by students in this educational level or to the more direct link with the labor market.

Spain has made significant efforts to create a high quality and equitable education system but there is still room for improvement. Developing positive classroom climates by enhancing positive teacher – student relationships and avoiding an emphasis on discipline alone could promote orderly, cooperative classroom environments more conducive to learning (Organisation for Economic Cooperation and Development [OECD], 2012). Teachers’ professional teaching experience, gender, and the educational level they teach should be considered as guidelines to provide a varied and tailored support linked to the priorities teachers have in different kinds of contexts or schools. This would enable the creation of an environment where teachers could feel supported and informed.

## Limitations of Present Study

This study took place in Spain with quite large samples, although the samples were drawn from a few regions only. Therefore, it would be desirable to extend the research to other Spanish regions in order to investigate the validity of the conclusions with a wider national sample. It would also be interesting to perform comparative analyses with other countries and cultural contexts to shed more light on the specific and generic roles of personal and contextual factors in explaining differences in teaching behavior quality. This limitation may help understand the low loads revealed in the regression analysis. Furthermore, the investigation of teachers’ teaching skills should consider more factors beyond educational level, teaching experience, and gender, involving more school and social contexts, teachers’ background, existing training programs or students’ engagement. All these points are the agenda for future research of the participating countries involved in this international project.

Moreover, the students participated in this study on a voluntary basis. All students who were present in the classroom when the questionnaires were distributed responded to the survey. Consequently, students who were absent (i.e., sickness, other reasons) did not participate. Future research should attempt to increase the number of participants and randomly sample students. It should be noted that all these results were based on student opinions. In other phases of the research it would be a good idea to compare these results with observations and teachers’ opinions about their professional practices in order to get a more accurate picture of the six teaching behavior domains and to triangulate information gathered by different methods such as classroom observations by external observers. Additionally, it would be worthwhile to empirically explore teachers’ beliefs about teaching as an important way to understand their behaviors.

Finally, some of the categories dealing with teaching experience are too broad due to the arbitrary and more practical reasons of categorization. In future research it may be necessary to establish more precise categories which may give more detailed information concerning possible differences in teaching behavior given teachers’ teaching experience in the classrooms.

## Author Contributions

C-MF-G, MI-C, and OG-P contributed to the implementation of the research in Spain, to the analysis of the results and to the writing of the manuscript. RM and MH-L designed and directed the study and also contributed to the analysis and writing of the manuscript. All authors discussed the results and commented on the manuscript.

## Conflict of Interest Statement

The authors declare that the research was conducted in the absence of any commercial or financial relationships that could be construed as a potential conflict of interest.
